# Transcriptomic Analysis of Salt-Stress-Responsive Genes in Barley Roots and Leaves

**DOI:** 10.3390/ijms22158155

**Published:** 2021-07-29

**Authors:** Rim Nefissi Ouertani, Dhivya Arasappan, Ghassen Abid, Mariem Ben Chikha, Rahma Jardak, Henda Mahmoudi, Samiha Mejri, Abdelwahed Ghorbel, Tracey A. Ruhlman, Robert K. Jansen

**Affiliations:** 1 Laboratory of Plant Molecular Physiology, Center of Biotechnology of Borj Cedria, B.P. 901, Hammam-Lif 2050, Tunisia; rimnefissi@gmail.com (R.N.O.); mariam.tn@gmx.fr (M.B.C.); rjardak@yahoo.fr (R.J.); mejrisamiha2018@gmail.com (S.M.); wahidghorbel@yahoo.fr (A.G.); 2 Center for Biomedical Research Support, University of Texas at Austin, Austin, TX 78712, USA; darasappan@austin.utexas.edu; 3 Laboratory of Legumes and Sustainable Agrosystems, Center of Biotechnology of Borj Cedria, B.P. 901, Hammam-Lif 2050, Tunisia; abidghassen@gmail.com; 4 International Center for Biosaline Agriculture, Dubai 00000, United Arab Emirates; HMJ@biosaline.org.ae; 5 Department of Integrative Biology, University of Texas at Austin, Austin, TX 78712, USA; truhlman@austin.utexas.edu; 6 Biotechnology Research Group, Department of Biological Sciences, Faculty of Science, King Abdulaziz University (KAU), Jeddah 21589, Saudi Arabia

**Keywords:** *Hordeum vulgare* L., salinity, RNA-seq analysis, differentially expressed genes, tolerance, candidate genes

## Abstract

Barley is characterized by a rich genetic diversity, making it an important model for studies of salinity response with great potential for crop improvement. Moreover, salt stress severely affects barley growth and development, leading to substantial yield loss. Leaf and root transcriptomes of a salt-tolerant Tunisian landrace (Boulifa) exposed to 2, 8, and 24 h salt stress were compared with pre-exposure plants to identify candidate genes and pathways underlying barley’s response. Expression of 3585 genes was upregulated and 5586 downregulated in leaves, while expression of 13,200 genes was upregulated and 10,575 downregulated in roots. Regulation of gene expression was severely impacted in roots, highlighting the complexity of salt stress response mechanisms in this tissue. Functional analyses in both tissues indicated that response to salt stress is mainly achieved through sensing and signaling pathways, strong transcriptional reprograming, hormone osmolyte and ion homeostasis stabilization, increased reactive oxygen scavenging, and activation of transport and photosynthesis systems. A number of candidate genes involved in hormone and kinase signaling pathways, as well as several transcription factor families and transporters, were identified. This study provides valuable information on early salt-stress-responsive genes in roots and leaves of barley and identifies several important players in salt tolerance.

## 1. Introduction

Salinity is one of the most pressing abiotic stressors threatening plant growth and agricultural production worldwide. Saline conditions are increasing rapidly along with the alarming rise of global warming, particularly in arid and semiarid regions [1]. Given these severe conditions, understanding the molecular mechanisms underlying salinity stress response in plants could contribute to the development of salt-tolerant crops in order to sustain productivity and quality.

Salinity is often recognized as an excessive accumulation of sodium ions in the soil [2], leading to osmotic stress and ion toxicity [3,4]. These two main effects of salt damage result in decreased photosynthetic efficiency, redistribution of cell wall constituents, reduction of cell expansion and division, and oxidative damage from reactive oxygen species (ROS) [2,5,6]. Hence, salinity stress generates deleterious effects on plant growth and productivity.

In response to salt stress, plants activate several tolerance mechanisms, including physiological, biochemical, and molecular changes. These diverse mechanisms allow the accumulation of osmoprotectants, regulation of ion homeostasis, and detoxification by the activation of ROS scavengers via efficient signal transduction networks [1]. Several important mechanisms have been characterized, including the osmoprotectant pathway and scavengers that regulate ROS homeostasis [7]. Both antioxidant enzymes and nonenzymatic compounds play critical roles in detoxifying ROS induced by salinity stress [8]. In addition, membrane transporters and ion channels, namely, the high-affinity potassium transporter and salt overly sensitive families involved in Na^+^-specific transport, play a crucial role in Na^+^ homeostasis through the regulation of K^+^/Na^+^ and H^+^/Na^+^ balances, respectively [4,9,10]. Further, several transcription factor families, such as the dehydration-responsive element-binding protein (CBF/DREB) family [11] and the mitogen-activated protein kinase family, function in pathways that regulate the expression of stress-related genes [12,13] along with other factors that regulate abiotic stress responses. Finally, the interaction of several plant hormones, such as abscisic acid (ABA), cytokinins, auxins, ethylene, salicylic acid, and jasmonic acid, plays vital roles in salt stress signaling and response [14,15].

High-throughput RNA sequencing (RNA-seq) is an important approach to study the expression of a large number of genes in a given tissue at a given time point [16]. RNA transcript profiling is a powerful technology for genomewide transcript characterization, differential gene expression analysis, variant detection, and gene-specific expression. These features are facilitating a deeper understanding of the genetic variation in complex phenotypic traits, such as salt tolerance, and allowing the enrichment of salt stress response pathways [17]. Several RNA sequencing studies have examined salt stress responses in different plants, such as barley (*Hordeum vulgare* L.) [18], sweet potato (*Ipomoea batatas* (L.) Lam.) [19], rapeseed (*Brassica napus* L.) [20], and *Arabidopsis* [21]. These studies suggest the involvement of a substantial number of salt tolerance genes encoding oxidation–reduction processes and osmoprotectant metabolism, ion transport, heat shock proteins (HSPs), and hormone signaling. Furthermore, differentially expressed genes (DEGs) encoding several transcription factors and signal transduction components associated with salinity tolerance have been identified. However, a paucity of information is available regarding differences between expression profiles of shoot and root in salt-tolerant barley cultivars.

Barley is an important food, feed, and industry crop with economic significance worldwide [22]. Barley yields are seriously threatened by escalating levels of salinization due to the overall reduction of root and leaf growth. Indeed, salt stress first impacts the root system of plants by inducing osmotic stress, leading to ion toxicity effects due to nutrient imbalance in cytosol, decreasing turgor due to limits in leaf gas exchange and stomata closure, and increasing oxidative damage, all of which interfere with normal cell division and expansion, leading to lower growth and yield rates [23,24,25,26]. Barley is still considered a relatively salt-tolerant crop and an important model for investigations of plant responses to changes in salinity [24]. Barley nuclear genomes are characterized by robust genetic diversity, making it attractive for stress tolerance breeding. Barley landrace accessions harbor novel genetic resources; Boulifa is a Tunisian accession with high salinity tolerance [27,28]. Therefore, investigating salt tolerance mechanisms remains important for barley breeding programs and helps to identify key genes involved in salt tolerance. The identified candidate genes represent valuable resources for future genetic engineering studies in cereals as well as in other crops towards the development of new varieties with more salt-tolerant characters and would be exploited to establish efficient applied breeding plans.

In order to identify candidate genes, molecular functions, and biological processes involved in response to salinity, stress high-throughput RNA-seq was performed on the salt-tolerant Tunisian accession Boulifa. Leaves and roots were examined separately and at different time points following exposure to severe salt stress (200 mM NaCl) in order to deepen our understanding of the specific response of these tissues under different stress durations. The results improve the current understanding of salt stress response mechanisms in barley leaves and roots and can be applied to developing salt-tolerant cereals.

## 2. Results

### 2.1. Analyses of RNA-Seq Datasets

**Figure 1:**
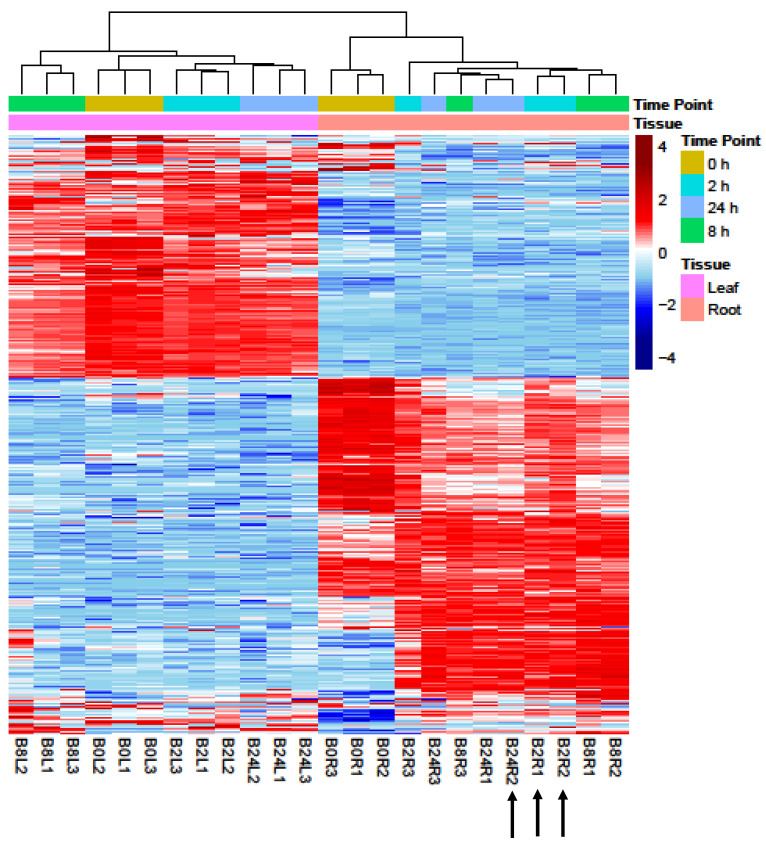
Cluster analysis using 25% highly variable genes. Gene expression in leaf (pink) and root (orange) was assessed under salt (200 mM NaCl) treatment for 2, 8, and 24 h relative to untreated plants (0 h). Samples and biological replicates (L1, L2, and L3 for leaves and R1, R2, and R3 for roots) are shown. The color gradient (upper right) indicates the pattern of expression from lowly (blue) to highly (red) expressed genes and ranges from −4- to +4-fold changes in expression. Sampling time point and tissue type are shown at the top of the cluster plot and defined on the right. The dendrogram at the top shows the sample clustering, and the black arrows indicate the samples that did not cluster by time point in the analysis.

An average of 23.9 million high-quality reads were obtained for each sample (an average total count of 24.14 million reads per sample before filtering low-quality reads (Table S1)). On average, 92.5% of the reads were mapped to the barley genome, indicating that the samples were comparable. On average, 62.5% of the reads were pseudoaligned to the barley transcriptome, and 32,587 genes were detected (Table S1). Furthermore, the time point clustering of replicates in 21 of the 24 samples, shown in Figure 1, indicates the high quality of sampling and RNA-seq analysis.

### 2.2. Differentially Expressed Genes in Leaves and Roots under Salt Stress

**Figure 2:**
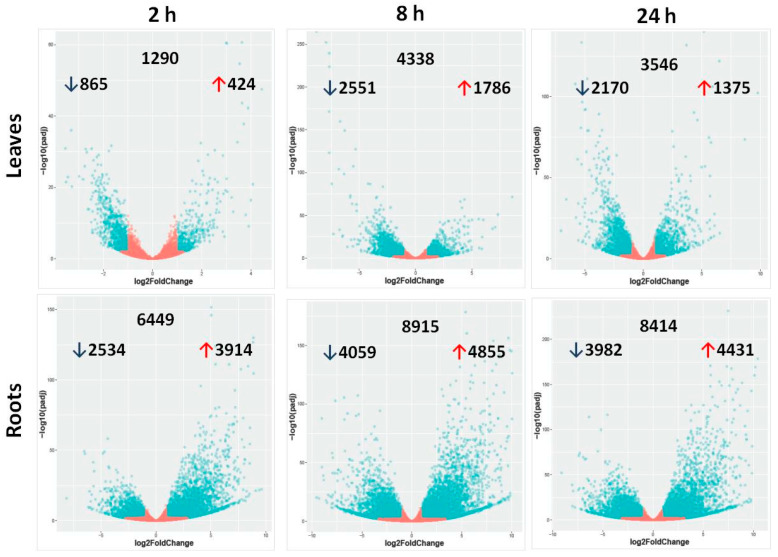
Volcano plots depict differentially expressed genes. Salt-treated plants were sampled at 2, 8, and 24 h of salt treatment, and differentially expressed genes (DEGs) were assessed relative to untreated plants (0 h) in leaves and roots. For each plot, the *X*-axis shows a log base 2-fold change, and the *Y*-axis indicates the adjusted *p*-values for the differences in expression. For each time point, the total DEG numbers are shown in the upper middle of each graph, upregulated DEGs are indicated by a red up arrow, and downregulated DEGs by a black down arrow. Blue dots and red dots equate to significant DEGs and nonsignificant genes, respectively.

**Figure 3:**
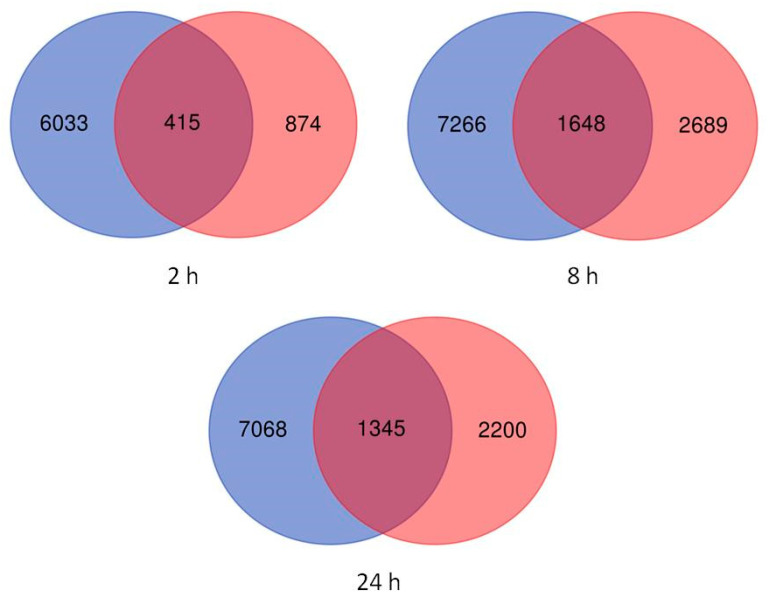
Shared and unique differentially expressed genes in barley seedlings. Differentially expressed genes (DEGs) exposed to 2, 8, and 24 h salt treatment were evaluated relative to untreated plants (0 h). Blue and red circles indicate roots and leaves, respectively, and shared DEGs are indicated by overlap. The number of affected genes is given for each segment of the Venn diagram.

Principal component analysis (PCA), a dimensionality reduction technique that projects high-dimensional data on the principal components that represent the largest variation in the data, was conducted in order to assess the largest source of variation among data. The samples were projected onto principal component 1 (PC1) as the *X*-axis and principal component 2 (PC2) as the *Y*-axis, representing, respectively, the first and the second largest sources of variation in the data. In Figure S2, PC1 (*X*-axis), which represents 86.5% of the variation in data, separates out samples by tissue, indicating that tissue is likely the largest source of variation in our data. Therefore, differential expression analysis was performed separately for each tissue. Differentially expressed genes were assessed in leaves and roots of plants exposed to short (2 h), intermediate (8 h), and long-term (24 h) salt stress, and responses were compared with pretreatment plants. The pattern of differential expression in untreated and salt-treated barley seedlings is shown in Figure 1. Red and blue signify overexpressed and underexpressed genes, respectively. The numbers of DEGs are depicted as volcano plots (Figure 2). For all salt treatment durations, differences in total DEGs were observed between leaves and roots. In leaves, the numbers of DEGs were 1290, 4338, and 3546 compared with 6449, 8915, and 8414 in roots after 2, 8, and 24 h salt treatment, respectively. The intermediate term response (8 h) showed the highest number of DEGs, followed by the long-term (24 h) and the short-term (2 h) (Figure S1). In both leaves and roots across time points, the greatest numbers of shared DEGs were between 8 and 24 h. Stress-responsive DEGs were either common to both tissue types or specific to each type (Figure 3). Tissue-specific DEGs were much more prominent in roots at all time points, particularly in short-term stress response. At 2 h of salt treatment, 6033 DEGs (representing 82% of the total DEGs in this tissue/time of treatment) were specific to roots, whereas only 874 DEGs (12%) were specific to leaves, and 415 DEGs (6%) were shared by both tissues. Intermediate- and long-term responses shared similar proportions of tissue-specific DEGs, with 63% and 66% DEGs in roots, 14% and 13% shared DEGs, and 23% and 21% DEGs in leaves at 8 and 24 h of salt stress, respectively.

At all time points, fewer up- than downregulated DEGs were detected in leaves (3585 up and 5586 down) (Figure 2). However, in roots, more DEGs were up- than downregulated (13,200 up and 10,575 down) (Figure 2). Across time points, only 921 DEGs (representing 0.36% of the total DEGs in leaves and roots) were oppositely modulated in the two tissues. The highest degree of differential modulation was observed at 8 h of salt treatment (501 DEGs) compared with only 69 at 2 h and 351 at 24 h of salt stress.

### 2.3. Gene Ontology Enrichment Analysis of Differentially Expressed Genes

**Figure 4:**
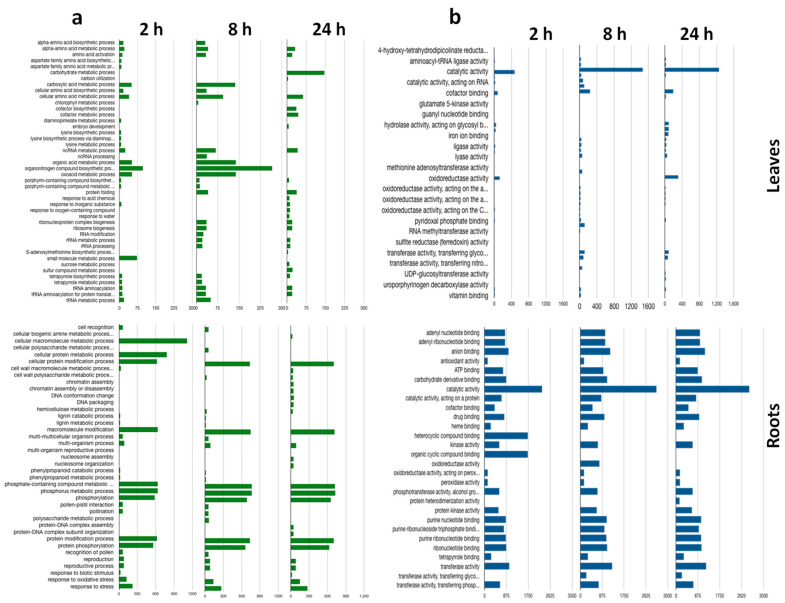
Gene ontology analysis. The gene ontology analysis of biological processes (**a**) and molecular functions (**b**) for the functional groups most significantly enriched among the differentially expressed genes (DEGs) under 2, 8, and 24 h salt stress in both leaf and root tissues. Gene ontology term categories are indicated on the *Y*-axis. Green (**a**) and blue (**b**) histograms indicate numbers (*X*-axis) of DEGs enriched in each category.

In leaf samples, the most enriched biological processes were biosynthetic and metabolic processes (Figure 4). These categories were consistent across time points, although the largest DEG numbers for both GO terms were detected at 8 h. In roots, the predominant processes were metabolic, cellular metabolic, and response to stress (Figure 4a).

Enrichment of GO terms involved in metabolism, such as biosynthetic, small-molecule, cellular, organic substance, nitrogen compound, and primary metabolic processes, was detected in both leaves and roots. Although these categories were enriched in both tissues, roots also included more enriched GO terms and several other metabolic processes, including cellular protein, lignin, and polysaccharide, along with protein modification and phosphorylation. Biosynthetic processes, such as alpha amino acid, cellular amino acid compound, lysine, and organic substance biosynthetic, as well as the chlorophyll metabolic process, were enriched only in leaves mainly after 8 and 24 h salt treatments. Response to stimulus, enriched predominantly in roots, included response to oxidative stress and response to biotic stress.

Catalytic activity was over-represented in both leaves and roots at all time points (Figure 4b). While this was the main molecular function highly enriched in leaves, several other GO categories were ascribed to roots, such as binding, antioxidant, and kinase activities. In both tissues, catalytic activity was mainly represented by GO terms for oxidoreductase, ligase, transferase, hydrolase, and decarboxylase. In roots, GO terms associated with binding affinity were mainly represented, such as nucleotide, protein, ion, organic cyclic compound, anion, carbohydrate, and ATP (Figure 4b).

KEGG (Kyoto Encyclopedia of Genes and Genomes) pathway analysis showed that the pathways enriched in leaves and roots in response to salt stress were different, but were conserved across time points for each tissue (Table 1). In leaves, porphyrin and chlorophyll metabolism, biosynthesis, and metabolism of various amino acids (lysine, alanine, aspartate, and glutamate), as well as biosynthesis of aminoacyl-tRNA, antibiotics, and several secondary metabolites, were identified. The over-represented pathways in roots were drug metabolism of various enzymes (oxidoreduction and phospholipase) and biosynthesis of phenylpropanoids, characterized by their antioxidant activity (such as phenylalanine and flavonoids).

### 2.4. Candidate Salt-Responsive DEGs

Based on functional annotation, several candidate genes were found to be differentially regulated in both leaves and roots at short- (2 h), intermediate- (8 h), and long-term (24 h) salt stress treatments. The most prevalent salt-responsive genes were categorized in different families, including ion-transporter-related, antioxidant, hormone-related, abiotic stress-responsive, transcription factors, and signal transduction (Table S2).

Several components of the ABA signaling pathway, such as ABA sensor pyrabactin resistance 1, ABA-independent SNF1-related protein kinase 2 (*SnRK2*), and 2C-type protein phosphatases (*PP2C*), as well as ABA-responsive elements GRAM-domain-containing protein and ABC transporter G family member 3, were differentially regulated under all stress durations in both tissues. Auxin-signal-transduction-related genes were also differentially expressed, including several auxin-responsive factors, auxin-induced proteins, and dormancy/auxin-associated family protein. Additionally, a number of both ethylene- and jasmonic-acid-mediated signaling pathways were differentially regulated in barley seedlings (Table S2).

Several other gene families involved in signaling were differentially regulated in both leaf and root tissues under all salt stress durations (2, 8, and 24 h), including calcium signaling, leucine-rich repeats receptor-like kinase (*LRR-RLK*), and protein kinases. Differential expression of several others was observed only in roots, such as the NTPase-domain- (*NACHT*) and the pyrin-domain (*PYD*)-containing protein. Among protein kinases, histidine kinase, histidine-tRNA ligase, and hybrid signal transduction histidine kinase I were identified. Compared with leaves, more differentially expressed kinases were detected in roots. Various transcription factor families were differentially expressed in both tissues, among them a basic helix-loop-helix DNA-binding superfamily protein, *WRKY* DNA-binding protein, kinase interacting (*KIP1-like*) family protein, homeobox-leucine zipper protein 3, and heat shock transcription factor C1.

Several oxidoreductase, glutathione S-transferase, and peroxidase families were differentially expressed, particularly in roots after 8 and 24 h salt exposure. Superoxide dismutase (SOD) and catalase antioxidant enzymes were also differentially regulated by salt stress. Catalase genes, including catalases 1 and 3, were upregulated in both leaves and roots under all stress durations except 24 h in roots, while Fe superoxide dismutase 2 was downregulated only in roots. Chalcone synthase 2, with a crucial role in ROS detoxification, was differentially expressed in both roots and leaves. Genes involved in the biosynthesis of proline, sugars, and glycine betaine, all of which are major osmoprotectants, were also differentially expressed in both tissues, particularly in leaves after 8 and 24 h salt stress. The ATP-binding cassette (*ABC*) transporter family, such as ABC transporter G family members, and solute transporters, including those for sugars, amino acids, and peptides, were differentially regulated in both tissues under all salt stress durations.

Transcript expression of various membrane transporters and ion channels, including glutamate receptor-like, cyclic nucleotide-gated, high-affinity K^+^ transporters (*HKTs*), salt overly sensitive Na^+^/H^+^ exchanger (*SOS*), and two-pore-domain K^+^ channel (TPK), were differentially expressed in both tissues mainly after 8 and 24 h salt treatments.

Components of photosystems I (*PSI*) and II (*PSII*) and light-induced proteins were also differentially expressed in both leaves and roots under all salt stress durations. Furthermore, plant–pathogen interaction genes, such as thaumatin superfamily proteins, and defensin genes were up- or downregulated.

### 2.5. Validation of RNA-Seq Data by Quantitative Real-Time qRT-PCR 

**Figure 5:**
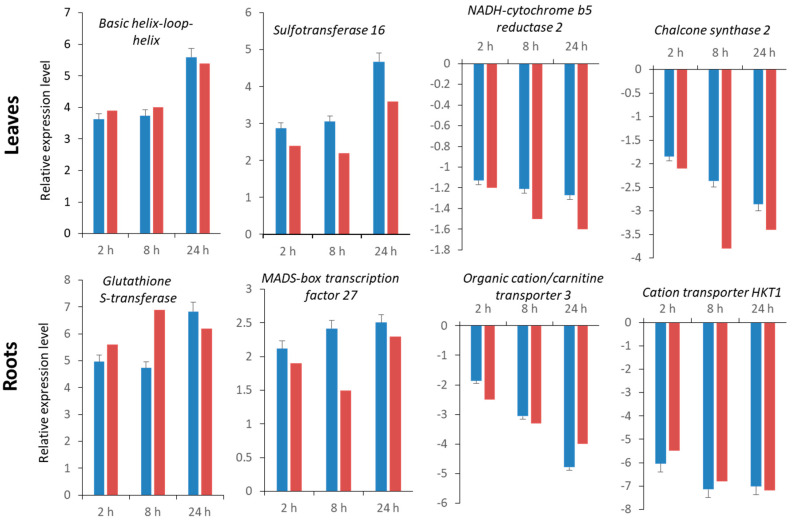
Expression pattern validation of eight randomly selected genes in leaves and roots by qRT-PCR. Red bar indicates transcript abundance changes of RNA-seq data calculated by the Log_2_ fold change method. Blue bar, mean with associated standard error bar (*n* = 3), represents the relative expression level determined by qRT-PCR using the 2^−ΔΔCT^ method.

Eight genes, including two up- and two downregulated genes in leaves and two up- and two downregulated genes in roots, were selected for confirmation of RNA-seq data by qRT-PCR. The expression fold changes for all six transcripts were in agreement with RNA-seq regardless of salt stress duration (Figure 5).

## 3. Discussion

Salt stress tolerance is determined by several interconnecting effects of different molecular, cellular, metabolic, and physiological mechanisms [8]. Understanding the networks that underlie the barley salt stress response will be of great interest for the identification of possible breeding targets in order to improve barley stress tolerance under a future scenario of global climate change.

Transcriptomic approaches can provide relevant information to elucidate the complex molecular and genetic mechanisms involved in barley salt tolerance response [17,29]. Furthermore, comparing transcriptomes of stressed vs. nonstressed barley plants across different time points supplies important key markers to support salt tolerance breeding programs. Tunisian local barely accessions may hold genes of high value for salinity tolerance due to their potential to grow under adverse conditions [28].

**Figure 6:**
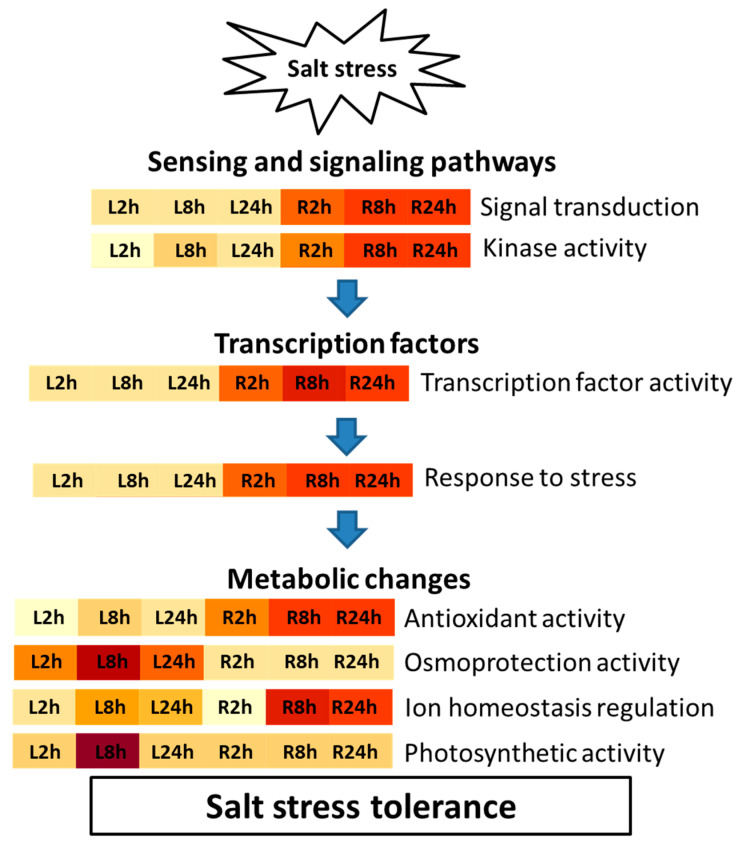
Flow chart of the salt stress tolerance mechanism in barley. The induced stress response begins with the activation of several sensing and signaling pathways, followed by transcriptional reprograming, resulting in the activation of diverse cellular homeostasis mechanisms for ROS detoxification, osmoprotection, ion homeostasis, and photosystem protection. Samples L2h, L8h, and L24h and R2h, R8h, and R24h for leaves and roots, respectively, under different stress durations are shown. The color gradient indicates the pattern of GO term enrichment level from low (yellow) to high (brown).

Barley leaf and root transcription profiles after 2, 8, and 24 h of high salt stress treatments (200 mM NaCl) revealed an array of up- and downregulations in various biological processes involved in the overall salt stress response (Figure 4 and Figure 6). Based on pairwise comparisons between control and salt-stressed samples, the number of DEGs was high and varied with the duration of salt treatment (Figure 2). Elevation of DEGs at all time points suggests important changes in the Boulifa seedling gene expression in response to salt stress. This extensive genetic regulation could be responsible for salt tolerance in the Boulifa genotype by affecting several physiological and biochemical processes. High salt induced the greatest number of DEGs following 8 h of exposure (Figure 2 and Figure S1). In agreement with a previous study, higher DEGs in wild barley leaves were found after 12 h of salt treatment compared with 24 h [30]. The salt-induced increase and subsequent decrease of DEGs may be attributed to the increasing stress of extended salt exposure, followed by possible recovery after 24 h. Based on these findings, 8 to 12 h of salt treatment should be the most appropriate duration to elucidate the genes involved salt stress responses. In contrast, a positive correlation between the duration of imposed stress and the number of DEGs in barley roots exposed to 6 and 24 h salt stress was demonstrated [15]. This discordance could be attributed to the differences in salt exposure times and/or imposed salt concentrations. Indeed, in our experiment the severe salt stress applied (200 mM) could induce a rapid and strong response compared with 150 mM used by Osthoff et al. [15].

Comparison of DEGs between root and leaf tissues revealed significant differences in expression in response to salt stress (Figure 1). For all treatment durations, the most severe impact on gene expression regulation was observed in roots compared with leaves (Figure 2, Figure 3 and Figure S1), emphasizing the more prominent role of roots in sensing salinity and responding through regulation of very complex transcriptional processes [31]. Early (2 h) salt-responsive DEGs were seven times more abundant in roots than in leaves; however, after 8 and 24 h, root DEGs were around three times greater than those of leaves (Figure S1), indicating a rapid salt stress response in roots, the primary organ of exposure [31]. At all time points, downregulated genes were more abundant than upregulated genes only in leaves. These results are consistent with previous reports on transcriptional responses in root and leaf tissues of different plant species subjected to abiotic stresses. Baldoni et al. [32] detected a higher number of DEGs in the roots (6007 genes) of a tolerant rice genotype (Eurosis) subjected to osmotic stress compared with leaves (3065) after 3 h treatment; however, the number of DEGs in both roots and leaves were similar after 24 h. Additionally, they detected a higher number of upregulated genes (61.6% of all DEGs) than downregulated genes (38.4%) only in roots. Furthermore, Luo et al. [19] detected more DEGs in roots than in leaves of salt-stress-treated sweet potato with also a greater number of upregulated DEGs than downregulated DEGs (544 up and 392 down) in roots and more downregulated DEGs than upregulated DEGs (75 up and 145 down) in leaves. Even in quinoa and peach, similar trends of DEG distribution between roots and leaves subjected to salt stress were reported [33,34]. The high number of downregulated genes in leaves under high salinity could be attributed to the efficiency in conserving resources and energy under stress conditions by repressing the transcriptional process of genes mainly associated with oxidative activities and cell wall compartment, which could be constitutively active. This may have contributed to the salt tolerance phenotype of Boulifa [28].

To gain further insight into the mechanisms underlying barley salt stress tolerance at an early seedling stage, DEGs in both leaves and roots were annotated, GO-enriched, and categorized into different functional groups (biological processes), including sensing and signaling pathways, transcriptional reprograming, hormone and ion homeostasis regulation, and metabolic changes as summarized in Figure 6.

To avoid salinity damage, plants have evolved sensors to detect stress and activate signal transduction for the modification of cellular traits through transcriptional regulation [35,36]. Therefore, sensing and signaling are crucial for salt stress response [37,38,39]. Several salt-induced signaling pathways have been previously reported, including abscisic acid (ABA) [40], hormone [41], calcium [42], and receptor-like kinase pathways [43] (Table S2).

The current study suggests the involvement of several signal transduction genes, including differentially expressed ABA signaling pathway genes. The ABA receptors, which are critical for plant growth and development under abiotic stress [44], were differentially expressed particularly in roots. Indeed, the ABA sensor pyrabactin resistance 1, a negative regulator of the ABA-independent *SnRK2* and a selective inhibitor of the *PP2C* [45], and both *PP2C* and *SnRK2*, major negative regulators of ABA signaling [46], were differentially expressed at all time points in both tissues. Furthermore, a number of ABA-responsive elements were differentially regulated under all stress durations.

Several other hormones play important roles in barley salt signaling, including differentially expressed hormone-related genes, such as auxins, ethylene, and jasmonic acid.

Auxins play an important role in determining plant architecture and contribute mainly to cell elongation and division. Several auxin-responsive protein family members, auxin signaling pathway constituents, and dormancy/auxin-associated family proteins, involved in defense against virulent bacterial pathogens [47], were differentially expressed, suggesting their important roles in barley salt stress response.

Ethylene is involved in ion homeostasis, ROS detoxification, and salt stress tolerance in plants [48]. Several genes encoding ethylene signaling pathway proteins were differentially expressed in both tissues under all stress durations. Relative to leaves, the majority of ethylene-signaling-associated DEGs were found in roots. Moreover, after 2 h salt exposure, the ethylene-signaling-associated DEGs were found only in roots (Table S2), demonstrating the rapid salt stress response in these tissues.

A number of jasmonic-acid-mediated signaling pathways that regulate many developmental and defense mechanisms, including root growth inhibition and activation of antioxidant enzymes upon exposure to high salinity [37], were upregulated under all stress durations mainly in roots. In addition, several calcium–calmodulin signal transduction proteins were differentially expressed in barley seedlings, particularly in roots, emphasizing the importance of calcium signaling and related mechanisms in regulating transcriptional activity in response to salt stress [37].

Various transcripts for proteins involved in signal transduction, such as the LRR-RLK protein and scaffold protein families were differentially expressed under all salt stress durations. Additionally, several transcripts encoding signal transduction histidine kinases were salt-regulated in barley seedlings. Relative to leaves, more kinases were differentially expressed in roots. These results are in agreement with previous studies [29,30,34] and confirm the involvement of signal transduction in salinity tolerance in barley and highlight the more complex signaling regulation in roots, supporting the notion that roots are the primary sensors of salt stress.

At 8 h of salt exposure, there were higher numbers of kinase DEGs in both leaves and roots. Furthermore, several transcription factors regulating gene expression in different signaling pathways [49] were identified under severe salt stress. Similar transcription factor families were identified by RNA-seq analysis in a mutant barley line exposed to salt stress [18].

Several metabolic pathways that include numerous proteins were differentially expressed in barley leaves and roots under salt stress. Indeed, in response to ROS, synthesis of stress-related metabolites (hormone, multiple osmolytes, and cell wall components) and photosynthesis and metabolite and water transport systems were upregulated (Table S2).

The antioxidant defense system was strongly affected in barley seedlings under salt stress with over-represented DEGs corresponding to antioxidant and oxidoreductase activities (Figure 4). Antioxidant enzymes such as SOD and catalase were represented among DEGs. These genes, widely described as active in ROS homeostasis [37], were also indicated through RNA-seq analyses of barley roots, mutant barley, and wild barley subjected to salt stress [15,18,30]. Chalcone synthase 2, which plays diverse roles in cell protection and detoxification as ROS scavengers and osmoregulators [1,15,31], was differentially expressed in both roots and leaves.

Several genes involved in the biosynthesis of major osmotic components, including proline, sugars, glycine betaine, and polyamines, were differentially expressed. These osmoprotectants, which have very important roles in maintaining water uptake, membrane and protein protection, and stabilization against abiotic stresses [50], were also identified in mutant barley exposed to salt stress [18]. These results highlight the involvement of osmotic and oxidative homeostasis maintenance in preventing stress-induced ROS accumulation, resulting in high growth performance previously detected in the tolerant genotype Boulifa [27,28].

Several proteins belonging to the ATP-binding cassette (ABC) transporter family involved in the modulation of stomatal response to CO_2_ [51] were upregulated in leaves and differentially expressed in roots under all stress treatments. Additionally, solute transporters were highly differentially expressed at all time points in both roots and leaves, suggesting their involvement in the re-establishment of cellular osmotic homeostasis.

Cellular ionic homeostasis, which is an essential process for growth during salt stress [52], was also highly differentially expressed in barley seedlings under salt stress. Two major classes of nonselective cation channels permeable to potassium and calcium were involved in re-establishing ionic balance after defense action [53]. The *HKT* transporters were upregulated in leaves and roots at all time points, while the *SOS*, which contributes to ion homeostasis by transporting Na^+^ out of the cell [54], was downregulated only in leaves after 24 h of salt treatment. The *TPK* channel, activated by calcium with strong selectivity for K^+^ over Na^+^, is involved in intracellular K^+^ redistribution between different tissues and stomatal regulation by monitoring turgor pressure [55]. The expression of this transporter was downregulated in both tissues at all stress time points. Other transporters active in the root tissue, such as the stelar K^+^ outward rectifier (*SKOR*) that mediates the delivery of K^+^ to the xylem for root–shoot potassium allocation [56], and the transient receptor potential cation channel subfamily V member 6 (*Trpv6*) that mediates Na^+^ and Ca^2+^ influx [57] were differentially expressed. In roots, expression of both genes was upregulated after 24 h of salt exposure, emphasizing the importance of root function in water and nutrient uptake during salt stress.

Salt-stressed plants must respond to not only ionic and osmotic disruptions but also impaired photosynthesis [37]. A large number of genes involved in the protection of photosystems were differentially expressed in leaf samples mainly after 8 h of exposure to salt stress. The regulation of these photosystem genes could be the origin of the sustained high growth rate, maintenance of water use efficiency, and photosynthetic potential in the tolerant genotype Boulifa under severe salt stress [27,28]. Significantly upregulated were components of PSI and PSII, including PSI assembly protein and PSII reaction center proteins required for stability and/or assembly, PSI P700 chlorophyll apoproteins, PSI iron–sulfur center, PSII D2 protein, and PSII 10 kDa polypeptide essential for photochemical activities [58]. Almost all of these genes were downregulated in roots under all stress durations. Earlier observations based on transcriptome analysis are consistent with this finding [32,59]. Both studies found downregulation of several photosynthesis-related genes in rice roots under osmotic stress and wheat roots under low-phosphorus stress. It was suggested that the repression of these genes in roots may be related to energy conservation. Furthermore, some transcripts related to carbohydrates, fatty acids, protein metabolism, and biosynthetic processes were differentially expressed in both leaves and roots in order to mobilize an alternative source of energy because of the impairment of photosynthesis during salt stress [1].

A number of pathogenesis-related (*PR*) genes were also differentially expressed in barley seedlings following salt stress. Upregulation of the *PR* gene expression was noted in 3-day-old barley seedlings geminated and grown in 100 mM NaCl [34]. At 8 h of salt stress, the leaf expression of a pathogenesis-related thaumatin superfamily protein was downregulated, while the expression in roots was downregulated at all treatment durations. The expression of another PR protein, *Solanum tuberosum* pathogenesis (STH-2), was upregulated only in roots, and defensin genes 1 and 2 were downregulated at all time points in roots but not at the 2 h time point in leaves. These observations support the hypothesis that pathogenesis-related proteins are involved in plant responses to environmental stresses.

## 4. Conclusions

In this study, a comprehensive comparison of the gene expressions of barley leaves and roots after 2, 8, and 24 h high salt stress was performed. The results suggest cross-talk among diverse pathways in barley in response to salt stress. Differential response included a rapid regulation of several candidate genes related to hormone and kinase sensing and signaling, such as ABA-responsive elements, calcium signaling, LRR-RLK, and protein kinases, and several transcription factors mainly belong to the MYB, bHLH, HD-ZIP, WRKY, MADS-box, and NAC families. Moreover, differential regulation of antioxidant genes, genes involved in the biosynthesis of osmolytes, and transporter genes was observed. Both common and tissue-specific salt-responsive candidate genes identified here constitute valuable resources for plant breeders and for further omics studies in barley and other crops. Future research, such transgenic assay, complementation assay, and subcellular localization, is needed to further validate the functions of the identified genes in providing salinity tolerance to plants and the physiological mechanisms in which they are involved.

## 5. Material and Methods

### 5.1. Plant Material and Hydroponic Salt Stress Treatment

Seeds of a salt-tolerant Tunisian barley cultivar (Boulifa) [27,28] were surface-sterilized with 5% sodium hypochlorite solution for 5 min, then thoroughly rinsed with distilled water and germinated in the dark at 25 °C in Petri dishes with distilled water. After 5 days, germinated seedlings were transferred to an aerated hydroponic system containing half-strength modified Hoagland’s solution [60] under 16 h light at 22 °C. After 3 days of acclimatization, gradual salt stress was applied. NaCl concentrations were brought up to 200 mM by increments of 50 mM NaCl on the first and second day and 100 mM on the third day. Root and shoot tissues were sampled at 0 h (before adding the first 50 mM NaCl), then again at 2, 8, and 24 h after reaching a final concentration of 200 mM NaCl. Five plants in each time point were harvested, pooled, washed thoroughly and separated into roots and shoots, frozen in liquid nitrogen, and stored at –80 °C for RNA isolation. All experiments were performed in triplicate.

### 5.2. Total RNA Isolation and DNase Treatment

Total RNA was isolated from shoots and roots representing each time point using the ZR Plant RNA MiniPrep™ Kit (Zymo Research, Irvine, CA, USA). The quality and quantity of isolated RNAs were checked by agarose gel electrophoresis and spectrophotometrically using a BioPhotometer (Eppendorf BioPhotometer plus, Hamburg, Germany). Residual DNA was eliminated using a TURBO DNA-free™ Kit (Promega, Madison, WI, USA).

### 5.3. Sequencing

Library construction and sequencing were carried out at the Beijing Genomics Institute (BGI, Shenzhen, China) for the three replicates of each treatment using the Illumina NextSeq 500 platform. Single-end reads 50 bp in length were generated for each sample at an average of 24.14 million reads per sample using the oligoDT selection method. Low-quality reads and adaptor sequences were removed from all samples (clean reads).

All clean reads were deposited in the Sequence Read Archive (SRA) database in NCBI with accession number PRJNA715166.

### 5.4. Pseudoalignment and Transcript Abundance Analysis

The reads were pseudoaligned to the barley transcriptome [61] (barley reference from PGSB barley genome database 2017) using kallisto [62], and gene-level abundances were obtained. The abundances were normalized using DESeq2 [63] and principal component analysis (PCA, Figure S2), and hierarchical clustering was performed using the top 25% highly varying genes in order to examine the underlying structure of the data and to identify the largest sources of variance.

### 5.5. Differential Expression Analysis

Differential expression analysis was performed separately for each tissue (root vs. leaf). Within each tissue, DESeq2 [63] was used to model the gene abundances as a negative binomial distribution, and three pairwise contrasts were performed (2 vs. 0 h, 8 vs. 0 h, and 24 vs. 0 h). Genes with adjusted *p*-value (after Benjamini–Hochberg correction) ≤ 0.01 and absolute log_2_ fold changes ≥ 1 were reported as significantly differentially expressed in each contrast.

### 5.6. GO Enrichment Analysis and Visualization

For each contrast, gene ontology (GO) terms enriched among the differentially expressed genes were identified using topGO [64], an R package that provides various algorithms for calculating the statistical enrichment of GO terms among a list of genes. A classic Fisher’s enrichment test was performed using each list of differentially expressed genes to identify significantly enriched GO terms in the molecular function and biological process domains.

All barley genes were annotated using Blast2go (OmicsBox 2.0.10) [65] to identify KEGG pathway information. Fisher’s enrichment test was run, and over-represented pathways were identified using FDR cutoff 0.1.

### 5.7. Validation of RNA-Seq Findings by Real-Time PCR

Validation of RNA-seq findings was performed by reverse transcription PCR. Quantitative real-time PCR (qRT-PCR) was performed using Applied Biosystems Power SYBR Green qPCR Master Mix (Life technologies, Carlsbad, CA, USA) on 96-well plates using specific primers designed by the Primer 3 software (http://bioinfo.ut.ee/primer3-0.4.0/) [66]. The expression levels of eight randomly selected genes and the internal control alpha tubulin (TUB2) were checked using an Applied Biosystems thermal cycler. The primer names and sequences used for primer design are in Table S3. Each qRT-PCR reaction mixture (20 µL) contained 1 µL of fourfold diluted cDNA, 10 µL of PCR mixture (2 × SYBR Green buffer), 7 µL of water, and 2 µL of primers (10 ppM). The following thermal profile was used: 95 °C (10 min), 40 amplification cycles of 95 °C (30 s), 60 °C (60 s). Melting curves were obtained by slow heating from 65 to 95 °C at 0.5 °C/s and continuous monitoring of the fluorescence signal. Results were analyzed by the StepOne^TM^ software, v2.2.2 (Applied Biosystems, Foster City, CA, USA). Transcript abundance quantification was performed according to the 2^−∆∆CT^ method [67]. Fold change was calculated for the salt-treated seedlings relative to the untreated plants (0 h).

Total RNA isolation, quantification, and DNase treatment were performed as previously described. cDNA synthesis was performed from 1 µg of RNA using a GoScript™ Reverse Transcription System Kit (Promega) following the manufacturer’s instructions.

## Figures and Tables

**Table 1:** Over-represented KEGG pathways identified in barley leaves and roots under salt stress.

Leaves		Roots	
KEGG ID	Enriched Pathway	KEGG ID	Enriched Pathway
Ko00860	Porphyrin and chlorophyll metabolism	ko00980	Metabolism of xenobiotics by cytochrome P450
Ko00970	Aminoacyl-tRNA biosynthesis	ko00982	Drug metabolism—cytochrome P450
Ko00261	Monobactam biosynthesis (glutamate dehydrogenase (NAD(P)+))	ko00983	Drug metabolism—other enzymes (phospholipase)
Ko00300	Lysine biosynthesis	ko00940	Phenylpropanoid biosynthesis
Ko00250	Alanine, aspartate, and glutamate metabolism	ko00460	Cyanoamino acid metabolism
Ko00997	Biosynthesis of various secondary metabolites	ko00480	Glutathione metabolism
Ko00270	Cysteine and methionine metabolism	ko00400	Phenylalanine, tyrosine, and tryptophan biosynthesis
Ko00941	Flavonoid biosynthesis	ko00140	Steroid hormone biosynthesis
Ko00332	Carbapenem biosynthesis (NADH-quinone oxidoreductase subunit C)	ko00943	Isoflavonoid biosynthesis
Ko00906	Carotenoid biosynthesis		

## Data Availability

The data presented in this study are openly available in the Sequence Read Archive (SRA) database in NCBI under accession number PRJNA715166.

## Supplementary Materials

The following are available online at https://www.mdpi.com/article/10.3390/ijms22158155/s1. Figure S1. Differentially expressed genes in leaves (a) and roots (b). Differentially expressed genes (DEGs) at 2, 8, and 24 h of salt treatments relative to untreated plants (0 h) are shown. The specific DEG numbers for each time point and the shared DEGs between the different time points are shown by dots and lines, respectively. Figure S2. Principal component analysis (PCA) of the data showing the variation due to tissue. Table S1. Statistics of RNA-seq numerical data analysis in barley seedlings. B0L (control leaves), B0R (control roots), B2L (leaves salt-stressed for 2 h), B2R (roots salt-stressed for 2 h), B8L (leaves salt-stressed for 8 h), B8R (roots salt-stressed for 8 h), B24L (leaves salt-stressed for 24 h), B24R (roots salt-stressed for 24 h). Table S2. Candidate genes of barley in response to salt stress, yellow: downregulated; red: upregulated; blue: up- and downregulated genes (multiple genes). Table S3. Primers used for qRT-PCR to validate RNA-seq findings.
