# Supplementary material for: Transcriptomic Analysis of Salt-Stress-Responsive Genes in Barley Roots and Leaves
Source: Int J Mol Sci. 2021 Jul 29;22(15):8155. doi: 10.3390/ijms22158155 (PMC8348758; doi:10.3390/ijms22158155)
Supplement: Supplementary file 1 [file ijms-22-08155-s001.zip › Supplementary Figures.pdf]

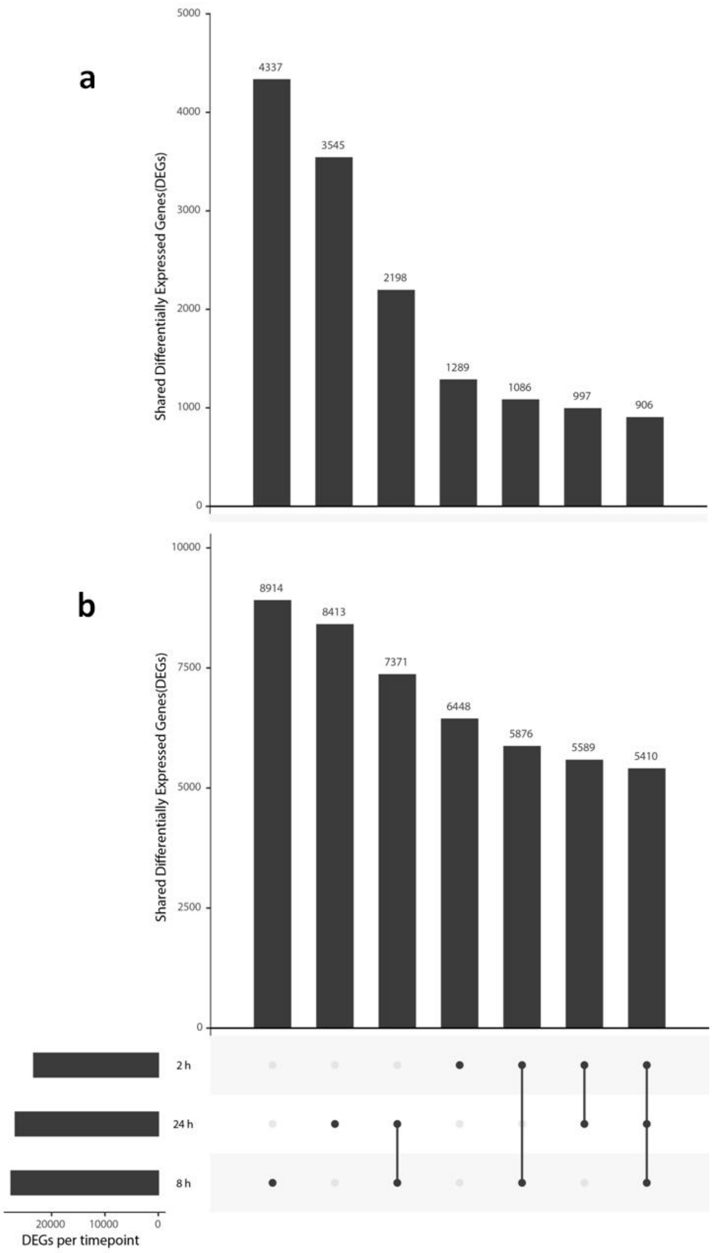

Figure S1. Differentially expressed genes in leaves (a) and roots (b). Differentially expressed genes (DEGs) at 2 h, 8 h, and 24 h salt treatments relative to untreated plants (0 h) are shown. The specific DEG numbers for each time point as well as the shared DEGs between the different time points are shown by dots and lines, respectively.

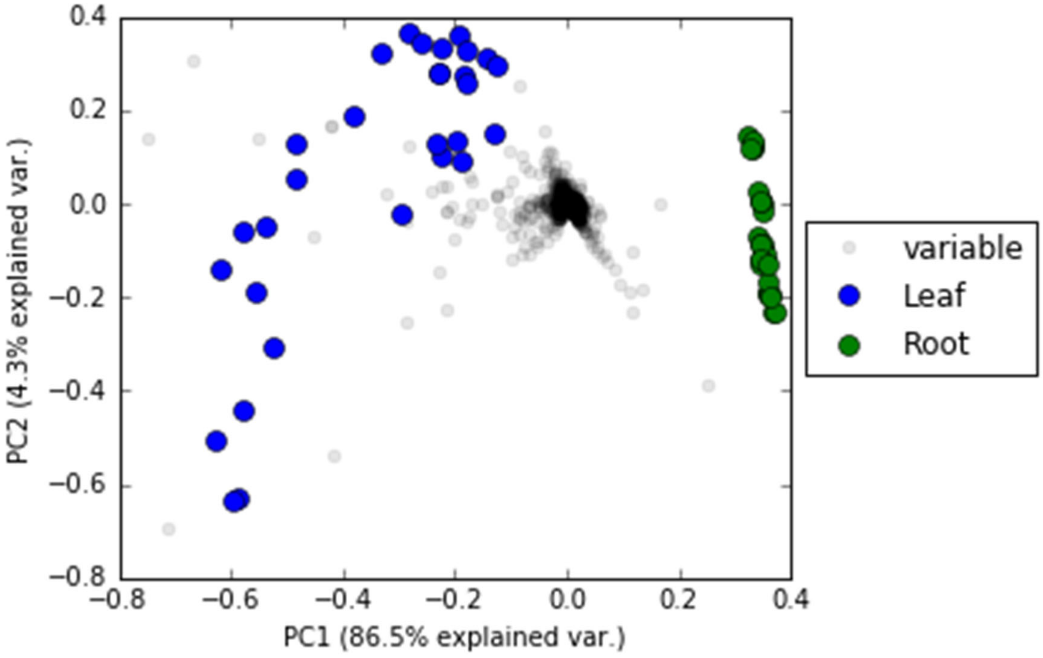

Figure S2. Principal component analysis (PCA) of the data showing the variation due to tissue.
